# Quantifying the effects of risk-stratified breast cancer screening when delivered in real time as routine practice versus usual screening: the BC-Predict non-randomised controlled study (NCT04359420)

**DOI:** 10.1038/s41416-023-02250-w

**Published:** 2023-04-01

**Authors:** D. Gareth Evans, Lorna McWilliams, Susan Astley, Adam R. Brentnall, Jack Cuzick, Richard Dobrashian, Stephen W. Duffy, Louise S. Gorman, Elaine F. Harkness, Fiona Harrison, Michelle Harvie, Andrew Jerrison, Matthew Machin, Anthony J. Maxwell, Sacha J. Howell, Stuart J. Wright, Katherine Payne, Nadeem Qureshi, Helen Ruane, Jake Southworth, Lynne Fox, Sarah Bowers, Gillian Hutchinson, Emma Thorpe, Fiona Ulph, Victoria Woof, Anthony Howell, David P. French

**Affiliations:** 1 NIHR Manchester Biomedical Research Centre, Manchester Academic Health Science Centre, Central Manchester University Hospitals NHS Foundation Trust, Manchester, England; 2 The Nightingale and Prevent Breast Cancer Centre, Manchester University NHS Foundation Trust, Manchester, M23 9LT England; 3 Manchester Breast Centre, Manchester Cancer Research Centre, University of Manchester, 555 Wilmslow Road, Manchester, M20 4GJ England; 4 Genomic Medicine, Division of Evolution and Genomic Sciences, The University of Manchester, St Mary’s Hospital, Manchester University NHS Foundation Trust, Oxford Road, Manchester, M13 9WL England; 5 Manchester Centre of Health Psychology, Division of Psychology and Mental Health, School of Health Sciences, University of Manchester, Coupland Street, Manchester, M13 9PL England; 6 Division of Informatics, Imaging and Data Sciences, School of Health Sciences, University of Manchester, Manchester, England; 7 Centre for Prevention, Detection and Diagnosis, Wolfson Institute of Population Health, Queen Mary University of London, London, England; 8 East Lancashire Hospitals NHS Trust, Royal Blackburn Hospital, Haslingden Road, Lancashire BB2 3HH Manchester, England; 9 NIHR Greater Manchester Patient Safety Translational Research Centre, University of Manchester, Manchester, M13 9PL England; 10 Patient representative, Manchester, England; 11 Research IT, IT Services, University of Manchester, Manchester, M13 9PL England; 12 Department of Medical Oncology, The Christie NHS Foundation Trust, Wilmslow Road, Manchester, M20 4BX England; 13 Manchester Centre for Health Economics, Division of Population Health, Health Services Research & Primary Care, School of Health Sciences, University of Manchester, Manchester, M13 9PL England; 14 Primary Care Stratified Medicine research group, Centre for Academic Primary Care, University of Nottingham, University Park, Nottingham, NG7 2RD England

**Keywords:** Genetics research, Health services, Epidemiology

## Abstract

**Background:**

Risk stratification as a routine part of the NHS Breast Screening Programme (NHSBSP) could provide a better balance of benefits and harms. We developed BC-Predict, to offer women when invited to the NHSBSP, which collects standard risk factor information; mammographic density; and in a sub-sample, a Polygenic Risk Score (PRS).

**Methods:**

Risk prediction was estimated primarily from self-reported questionnaires and mammographic density using the Tyrer–Cuzick risk model. Women eligible for NHSBSP were recruited. BC-Predict produced risk feedback letters, inviting women at high risk (≥8% 10-year) or moderate risk (≥5–<8% 10-year) to have appointments to discuss prevention and additional screening.

**Results:**

Overall uptake of BC-Predict in screening attendees was 16.9% with 2472 consenting to the study; 76.8% of those received risk feedback within the 8-week timeframe. Recruitment was 63.2% with an onsite recruiter and paper questionnaire compared to <10% with BC-Predict only (*P* < 0.0001). Risk appointment attendance was highest for those at high risk (40.6%); 77.5% of those opted for preventive medication.

**Discussion:**

We have shown that a real-time offer of breast cancer risk information (including both mammographic density and PRS) is feasible and can be delivered in reasonable time, although uptake requires personal contact. Preventive medication uptake in women newly identified at high risk is high and could improve the cost-effectiveness of risk stratification.

**Trial registration:**

Retrospectively registered with clinicaltrials.gov (NCT04359420).

## Background

Breast cancer is the most common cancer and a leading cause of female death in the UK, especially at younger ages [1]. Approximately 55,000 women are diagnosed with breast cancer annually, while ~11,400 succumb to the disease [1]. Although breast cancer deaths have been falling in many Western countries, the incidence of breast cancer continues to rise [2–4]. To identify breast cancer at earlier and potentially more curable stages, close to two million women are screened in the National Health Service Breast Screening Programme (NHSBSP) in England annually [5]. The NHSBSP currently invites all women aged 50–70 years for 3-yearly mammograms.

Comprehensive risk assessment in the NHSBSP could identify many of the 5 in 6 women who are at National Institute for Health and Care Excellence (NICE) for England & Wales defined high risk [6] but are not currently aware of this [7, 8], as well as an even larger number of women at moderate risk. We have previously shown it is feasible to accurately estimate women’s individual risk of developing breast cancer through self-reported answers to questions assessing family history, reproductive and hormonal factors. This can be done by using the Tyrer–Cuzick risk algorithm and information on breast density obtained from mammograms [9] and this is further substantially improved by adding in information from a polygenic risk score (PRS) using saliva DNA [10–13]. A previous population-based study (PROCAS-1) provided 10-year risk estimates to more than 57,000 women in the NHSBSP in Manchester, North-West England [10, 12]. This study was the first time that personalised breast cancer risk estimates were calculated for large numbers of women from the general breast screening population, with risk feedback occurring for most 3–5 years post enrolment. The PROCAS-1 study found that at least 3% of women were high risk (≥8% 10-year risk) when standard risk factors plus mammographic density were assessed and a further 10% were defined as moderate risk (5–7.9% 10-year risk) [7, 8, 12]. We have previously estimated that only 1 in 200 (0.5%) of the population have currently identified themselves as high risk [7, 8]. This means that there are approximately an additional 450,000 women in England at high risk that NICE guidance indicate could be offered breast-cancer-preventive-medication-(BCPM) from age 30 years and annual mammography aged 40–59 years.

NICE recommended in 2013 that women at high risk of breast cancer (lifetime risk ≥30%, 10-year risk ≥8%), should be offered annual breast screening between the ages of 40–59 years; and those at moderate risk (lifetime risk=17–29%, 10-year risk=3–7.9% aged 40 years), should be offered annual mammograms from 40–49 years [6], and considered for annual mammography aged 50–59 years. NICE guidance also recommends that women at high risk of breast cancer are offered BCPM with tamoxifen, anastrozole or raloxifene (considered in moderate risk) along with advice on weight control and physical activity [6]. Thus far, it is estimated that only about 1 in 6 women who are at high risk as defined by NICE have been actively identified by attending Family History, Risk and Prevention Clinics (FHRPC) [7, 8].

We developed a partially automated system (BC-Predict) for offering breast cancer risk assessment and stratification to women when they accept their invitation for an NHSBSP mammogram, which included a risk factor questionnaire, volumetric mammographic density analysis and feedback letters of risk level to both the women and their primary care practitioners. A development phase involved working with healthcare professionals to ensure that the care pathways were workable and achievable [14] and working with women from British-Pakistani origins to identify likely barriers for them to access this system [15]. We checked that informatics procedures functioned as desired. Participant information materials based on the PROCAS-1 materials were co-developed with women who would be eligible for BC-Predict to promote good understanding and informed choices, and to minimise any harms such as worry [16].

The introduction of risk stratification in the NHSBSP could facilitate the potential benefits of more frequent annual mammography (potentially lowering tumour stage) and/or preventive medication in those at increased risk to be realised on a population basis. This would also potentially allow women at lower risk to have less frequent mammography. In principle, a risk-stratified NHSBSP should result in a better balance of benefits, harms and costs to the NHS and there is some early evidence to support this premise [17, 18]. The benefits include fewer breast cancers from use of BCPM, and potential reduced breast cancer mortality from the NHSBSP detecting a higher proportion of breast cancers at an earlier and more curable stage. There might also be a justification for reducing the intensity of screening for women at lower risk, who would be less likely to develop high-stage tumours and have lower-grade invasive tumours and more potential overdiagnosis as a proportion of tumours identified [11].

The consequences of actually introducing risk stratified screening as part of routine practice in the NHSBSP remain unclear. In the PROCAS-1 study, communication of risk estimates occurred 3–5years after women provided their consent and questionnaire information [10]. Therefore, that study provided limited information about the consequences of receiving risk estimates. It is probable that, if aware of their breast cancer risk, around 10% of women at high/moderate risk would opt for the NICE approved BCPMs (anastrozole/raloxifene/tamoxifen)- [7, 8, 10, 19, 20], and the majority for extra mammography in high risk women [8]. In contrast, there are also potential harms that could be caused by receipt of risk estimates, such as increasing anxiety and inequalities in uptake of screening. Although the best currently available evidence suggests this is unlikely, this evidence has limitations such as a long time-lag between women agreeing to risk assessment and receiving their risk results [20–22]. However, despite much ongoing work around establishing effectiveness of risk-stratified screening, there remains considerable uncertainty around uptake of these various offers in routine practice, which will impact on clinical and cost-effectiveness if this is implemented [23].

The present study aimed to quantify these likely benefits and harms of introducing risk-stratified screening in the NHSBSP [24]. Our objectives were to quantify uptake of BC-Predict amongst women offered it and potential benefits including uptake of risk consultation (for those eligible), BCPM (for those offered it) and additional mammography (for those offered it).

## Methods/design

### Study design

The present study (PROCAS-2) employed a non-randomised controlled trial of the effects of offering women either standard NHSBSP mammography screening or BC-Predict alongside the NHSBSP offer of mammography screening. BC-Predict involved obtaining breast cancer risk information from women to input into the computer model Tyrer-Cuzick (v8), incorporating an automatic measure of breast density (and in a small proportion a polygenic risk score from saliva DNA) to generate 10-year breast cancer risks for feedback. NHS ethical approval for the PROCAS-2 study was granted by Harrow Research Ethics Committee (ref 18/LO/0649)/IRAS-project ID:239199, and it was included in the ISRCTN registry, with fuller study details included in a study protocol paper [24]. All women involved with the PROCAS-2 study were invited from NHSBSP sites run by three services in North-West England. Women from five participating sites were also offered BC-Predict, and women from two other sites were only offered standard NHSBSP (with BC-Predict sites listed in Table 1).

Nested within the BC-Predict intervention arm was a further adaptive non-randomised factor, whereby various changes were made to methods of recruitment including changes to the information sheets and invitation letters and variations in uptake rates were noted (Supplementary Table 1).

After a pilot phase, BC-Predict started in September 2019 in two Greater Manchester sites (Trafford and Withington Community) Phase 1. The intended start was delayed in the two East Cheshire sites (Supplementary Table 1). A research practitioner was utilised from January to March 2020 to aid recruitment on site at Withington Community and to offer saliva sampling to generate a single-nucleotide polymorphism (SNP PRS). The study was seriously disrupted by the COVID-19 pandemic with a full shutdown of mammography occurring in England on March 21, 2020, and only reopening on 1 August 2020. It was not possible to offer saliva testing from this point until near the end of the study. Also screening invitations were severely curtailed due to needs for social distancing and enhanced cleaning of equipment after each woman was screened. From August 2020, the NHSBSP invitation process changed and women did not receive a pre-scheduled appointment for mammography, rather a letter inviting them to make an appointment by calling the relevant screening site.

Therefore, recruitment did not finish in the first two sites until September 2, 2020. The switch over (Phase 2) to Oldham in Greater Manchester, The East Cheshire sites and the two North-East Lancashire sites occurred on the 3 September, 15 September 2020 and 2 February 2021, respectively. The study was extended to July 9, 2021 at all Phase 2 sites to adjust for the reduced invitation rates for screening.

### Setting

Women were recruited from seven sites/vans (some static sites and some screened in mobile vans) within three NHS Breast screening programmes: three sites within the Greater Manchester breast screening programme (Withington Community Hospital, Oldham lntegrated Care Centre and the Trafford mobile screening van only), and two sites/vans each from the East Cheshire (Macclesfield District General Hospital and Stockport mobile breast screening van locations) and East Lancashire (Burnley General Hospital and North-East Lancashire mobile breast screening van locations) programmes.

BC-Predict risk estimation was undertaken via an online web system for women attending mammography screening. This included a risk assessment platform which facilitated consent and completion of a self-report questionnaire. Risk assessment and feedback (feedback within 8-weeks) was based on self-reported answers to the questions and breast density estimates automatically derived from raw mammography data using the Volpara^TM^ system. This was also able to incorporate information from currently known breast cancer Single-Nucleotide-Polymorphisms-(SNPs), obtained from DNA contained in saliva samples (initial target 1000 women) as a PRS. Women who received a negative result from their mammogram were then sent letters providing their 10-year breast cancer risk within 6–8 weeks after their mammogram allowing for them to have received the all clear from this. Those women at NICE defined moderate (>5% but <8% 10-year risk) or high risk (≥8% 10-year) were encouraged to attend a consultation at a FHRPC, to discuss the offer of annual screening and BCPM. The feedback letters contained the following text ‘We would encourage you to make an appointment with a doctor or nurse to discuss your risk further’ with contact details. Women were offered face to face or telephone appointments with an experienced risk provision clinician (AH, SJH, DGE) or nurse. As a result of COVID-19, only telephone appointments were offered after March 2020. NICE guidance states that tamoxifen only should be offered to pre-menopausal women, anastrozole as first line to post-menopausal with tamoxifen second and raloxifene as an alternative. Women were also allowed personal choice in BC-Predict in the post-menopausal setting. Healthy lifestyle and if appropriate weight loss measures were discussed at risk appointments and were signposted in feedback letters.

### Participants

Two groups of women were initially invited to participate in the study: women invited for their first mammogram (“prevalent screens”), and those women invited during the screening round within which they reach 60 years (“incident screens” i.e. women aged 57–63 years). This was expanded to women of any screening age in Phase 2. Social deprivation was assessed for each site by the mean Index of Multiple Deprivation which indicates area deprivation for England in deciles from 1 (most deprived) to 10 (least deprived) [25]. We have provided the national position in Table 1.

**Table 1:** Recruitment to BC-Predict by screening area.

Unit	Trafford	Withington Community	Oldham	East Lancashire	Macclesfield	Stockport	
Index of Multiple Deprivation^a^	209 of 371	2	29	11	228	130	–
Position rank in study	5	1	3	2	6	4	–
Number of women attending screening in 2021 or during study period^c^	2852	6828	4816	5307^c^	5738^c^	6757^c^	
Uptake to BC-Predict by screening area							
	Phase 1		Phase 2^b^				Total
Joined BC-Predict	445	405	256	280	510	576	2472
Invited	3750	6120	2149	2536	2247	2662	19464
% uptake	11.87%	6.62%	11.91%	11.04%	22.70%	21.64%	12.70%
Uptake screening overall	63%	53%	55%	53.40%	63.80%	63.80%	60.7%
Uptake of self-made mammography appointments	N/a	N/a	92.43%	93.12%	95.36%	96.31%	94.39%
Uptake of those attending screening	18.84%	12.49%	12.89%	11.86%	23.80%	22.47%	16.86%
Number attending screening	2363	3244	1986	2362	2143	2564	14661
Adjusted number of those invited for screening^b^	3750	6120	3907	4749	3521	4172	26219
Adjusted overall uptake^b^	11.87%	6.62%	6.55%	5.89%	14.48%	13.81%	9.42%
Odds ratio	1.79	Reference	0.99	0.87	2.19	2.09	
Odds ratio after removal of direct approaches	2.07	Reference	1.42	1.30	2.62	2.47	
*P* value	<0.0001	Reference	0.0003	0.846	<0.0001	<0.0001	

^a^Position rank from highest deprivation to lowest in English local councils.

^b^Adjustments required for overall uptake of BC-Predict in phase 2 to account for invites only going to those who made mammography appointments.

^c^Screening period 03/02/2021–09/07/2021 for East Lancashire and 15/09/2020–09/07/2021 for Macclesfield and Stockport.

Other inclusion criteria were that the participant: (1) was born biologically female; (2) was able to provide informed consent and (3) complete a risk assessment questionnaire. Exclusion criteria were that the participant: (1) previously had breast cancer; (2) had underwent a bilateral mastectomy; or (3) had previously participated in the PROCAS-1 study [10]. The decision to exclude PROCAS-1 participants (2009–2014) was reversed in Phase 2 as it was decided that enough time had elapsed to update risk information.

### Procedure

Women being offered BC-Predict were sent an invitation letter 1–2 working days after their breast screening invitation letter was sent. The BC-Predict invitation letter was sent along with the participant information sheet and instructions directing prospective participants to the online risk assessment platform. Each invitation letter included details of the participant’s “Date of first offered appointment”. This is the first breast screening appointment date that was offered to the participant. This date was relevant because participants could join the study either before the date of their first offered appointment or up to six-weeks after. After this time, it was not possible for them to login to the BC-Predict risk assessment platform.

Once participants consented to the study online via the online form after reading the participant invitation letter and information on consent online and completed the eligibility criteria, they were directed to the BC-Predict risk assessment questionnaire. Participants could answer part of the questionnaire, save, and return at a later date, as long as this occurred within their 6-week recruitment window. Assessment of the online questionnaire during the pilot phase (*n* = 150) estimated that most women would complete this within 20 min. If a prospective participant did not have access to the Internet, a paper version of the questionnaire could be posted out to be completed along with a paper version of the consent form. The data recorded on the questionnaire was then manually inputted into the online risk assessment platform by a study team member. No patient identifiable data was disclosed outside the study team to the online tool or Volpara^TM^ systems.

Once a negative mammography result was provided, risk feedback letters were generated by a member of the study team based on the participant’s answers on their questionnaire and mammographic breast density (calculated from uploaded raw data by Volpara^TM^ systems). The percentage density was inserted into an online version of Tyrer-Cuzick v8 that includes an algorithm to adjust density for age, BMI and menopausal status into an odds ratio known as density residual [12]. A sub-study (target=1000) also offered saliva testing for a PRS based on 143 SNPs as previously described [13]. This was added to the Tyrer–Cuzick algorithm as previously described via the tool function for PRS using an odds ratio [13]. The risk feedback letter informed women that they were at “high” (≥8% 10-year risk), “above average (moderate)” (≥5% but <8% 10-year risk), “average” (≥2% but <5% 10-year risk), or “below average” risk (<2% 10-year risk). Each letter explained how the risk estimates were derived, the factors that had contributed to the personalised calculation, the implications of the different risk categories and associated options to reduce their risk. An information leaflet further explaining risk factors and signs and symptoms of breast cancer was provided with the letter (supplementary information files). Letters to women at high or moderate risk recommended contacting the regional healthcare centre indicated to discuss further prevention/early detection options at an FHRPC.

To assess self-reported harms and benefits, and to inform a subsequent economic analysis, allied studies involved projected sub-samples of *n* = 2108 women (*n* = 1054 each from usual care NHSBSP and BC-Predict) who were invited to complete questionnaires assessing psychological benefits and harms of BC-Predict at baseline, 3-months and 6-months [26]. This study also used qualitative methods to understand the experiences of women who took part in BC-Predict and allied health professionals’ views of workloads and implementation [27].

### Measures

The following outcomes were assessed to determine the primary objective of feasibility of risk stratification:

1. Screening attendance at or within 180 days of the initial screening appointment
2. Uptake to BC-Predict of those attending screening
3. Time to provision of risk feedback letter and proportion over 8-week threshold
4. Subsequent consultation in FHRPC clinics (telephone as face-to-face not possible after March 2020)
5. Subsequent enrolment for more frequent mammography (NICE approved through FHRPC to age 60 or self-funded outside)
6. Subsequent prescription of BCPM.

### Analysis plan and sample size calculations

On the basis of PROCAS-1 results we anticipated that ~12% of women using BC-Predict in PROCAS-2 would have sufficient risk to be considered for BCPM and that approximately 10% of these would take it up [8, 28, 29]. Thus, 117 of the originally intended 8000 women (1.5%) receiving the intervention might be expected to be prescribed BCPM. It was anticipated that very few of those, if any, sent the standard screening invitation would be prescribed BCPM. Even if as many as 0.9% did so, we would still have had 90% power to detect this as a significant difference at the 5% level with two-sided testing, and 80% power compared 10% uptake through BC-Predict. The Greater Manchester Medicines Management Group agreed a shared care protocol stating that the initial prescription of tamoxifen and anastrozole should be made by a FHRPC specialist. As such, data from even those in the control arm should be available from prescriptions made in or letters or recommendation to prescribe sent from the FHRPC.

Two-sided chi square tests were used to assess differences in uptake between BC-Predict and standard NHSBSP controls using Fisher’s exact test (*P* < 0.05).

## Results

### Mammography attendance and BC-Predict uptake

**Fig. 1:**
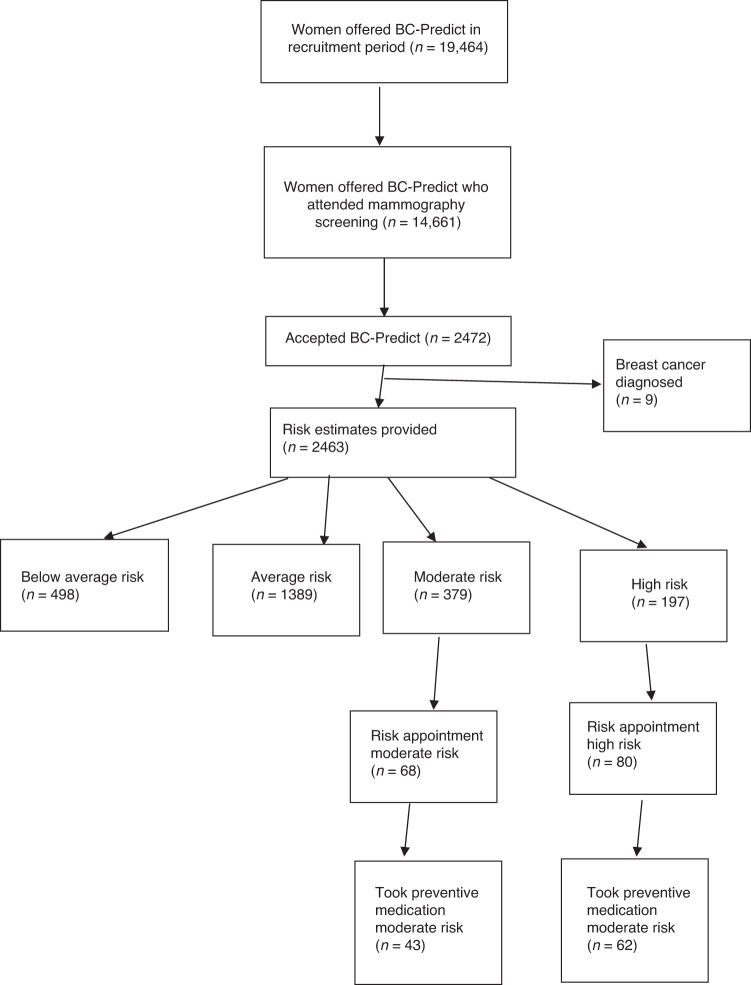
BC-Predict flow diagram. Showing the journey through BC-Predict of those invited and who consented to the study.

Attendance at mammography appointments at both BC-Predict and standard NHSBSP sites was lower than in the pre-pandemic era for COVID-19 (~70%) with only 60.7% mean attendance (Table 1 and Fig. 1). Uptake to BC-Predict (*n* = 2472) was 12.7% of those invited (adjusted to 9.4% when adjustments were made for Phase 2 as during this time, women made their own mammogram appointments) and 16.9% of those who attended for mammography. Overall uptake at the Withington Community study site was 12.5%. However, uptake was much higher in a sub-set of women directly approached face-to-face at this site (132/263, 50.2%; see Table 2). This was further enhanced by the offer of a paper questionnaire rather than using the online risk assessment platform to complete the questionnaire. Uptake increased to 79/125 (63.2%) despite overall Withington Community recruitment being only 12.5% of those attending screening (*P* < 0.0001). Indeed, excluding the 109 recruited on site who had not already consented to BC-Predict, uptake of those attending for screening without a direct approach was only 296/3244 (9.1%). Of those offered the paper questionnaire, 51/68 (75%) chose that option over using the online risk assessment platform.

**Table 2:** Uptake of BC-Predict in those personally approached at study site.

Women	All	Offered paper Questionnaire^a^
BC-P women attended (*n*)	305	145
BC-P women approached (*n*)	263	125
% of BC-P women approached	86.23%	86.21%
Women consented prior to clinic date (*n*)	23	16
% with prior recruitment	8.75%	12.80%
Women provided a saliva sample (*n*)	132	68
% of Women uptake to SNP	50.19%	54.40%
Overall recruitment to BC-Predict	52.00%	63.20%

^a^51/68 (75%) women offered paper questionnaire opted for paper.

Both screening attendance and BC-Predict uptake was highest in East Cheshire, which has a much lower mean deprivation score-(Table 1). Overall uptake of both BC-Predict and mammography screening was related inversely to the index of Multiple Deprivation Mean scores, see Table 1). There was a linear trend based on the rank position in the study and uptake with a Mantel–Haenszel chi square for linear trend=401.03; *P* < 0.0001. An exception was a lower uptake in Trafford in Phase 1 which may reflect teething problems with the online system and the original invitation letter. Uptake of those invited was higher in Phase 2 (*P* < 0.0001), most probably as women were only invited to BC-Predict if they made an appointment for mammography. Adjustments for this are made in Table 1 for overall uptake to the study if all eligible women had been invited given that in Phase 2, only those who made their own mammogram appointments were also invited to BC-Predict. Although uptake in Withington Community was initially higher than Oldham and East Lancashire, after adjusting for the direct face-to-face recruitment approach, this fell to only 9.1% as above. We were unable to assess uptake by ethnicity as the screening invitations do not have ethnicity recorded. Of 2347 (96.6%) women self-reporting ethnicity in the risk assessment questionnaire, 2020 (86%) were white British.

### Uptake of risk consultation appointment breast cancer preventive medicines and additional mammography

Attendance at risk feedback appointments was also lower than expected with 80/197 (40.6%) of high risk and 68/379 (17.9%) of women assessed at moderate risk taking up the offer of an appointment. However, the uptake of BCPM amongst women accepting a risk appointment was high (Table 3). Preventive medication was prescribed for 62/80 (77.5%) of women at high risk and 43/68 (63.2%) of those at moderate risk. The overall uptake of BCPM in those offered BC-Predict (105/19,464; 0.54%) was significantly higher than the zero uptake in the control (standard NHSBSP offer) arm (*P* < 0.0001). Overall uptake of BCPM from the intervention of BC-Predict in those consenting and receiving feedback was 105/2463 (4.3%). Of those prescribed BCPM, 89 were prescribed anastrozole, 13 tamoxifen and 3 raloxifene. There was also high uptake of opting for additional mammography at 78.8% in the high-risk and 64.7% in the moderate-risk women who had a risk appointment.

**Table 3:** Uptake of risk consultation appointments, breast cancer preventive medicines (BCPM) and additional mammography screening.

	High	Mod	Ave	Low	Total
*N*	197	379	1389	498	2463
%	8.00%	15.40%	56.40%	20.20%	100%
Risk appointment	80	68	1	0	149
%	40.60%	17.90%	0.07%	0.00%	6.05%
*P* value	*P* < 0.0001	Reference			
BCPM	62	43	0	0	105
% of all	31.50%	11.30%	0.00%	0.00%	4.26%
*P* value	*P* < 0.0001	Reference			
% seen	77.50%	63.20%	0.00%		70.47%
Additional screening NHS	46	11			57
Additional screening Private	17	33			50
Total	63	44			107
% uptake additional screening of those seen	78.75%	64.71%			71.81%

### Risk levels of the BC-Predict cohort

Overall risk levels suggested that a relatively high-risk population joined BC-Predict with 23.4% being actionable high risk (8.0%) or moderate risk (15.4%). However, the 227 who were recruited face to face at Withington Community (who provided a saliva DNA) had a lower proportion with only 11 (4.5%) high and 26 (11.45%) moderate risk. (Supplementary Table 2). The face-to-face group had a lesser degree of family history (lower rate of affected first degree relatives with breast cancer) than those only consenting to standard BC-Predict (without PRS). Particularly in Withington where family history of breast cancer in a first degree relative dropped from the overall average of 28% to 16.9% (*P* < 0.0001).

### Time to receiving risk feedback

For women not having a SNP-PRS, 76.8% were sent their BC-Predict risk assessment letter within 8 weeks of attending for mammography (Table 4). In Phase 2 of BC-Predict in Greater Manchester, results delivery improved to 97.4% in Oldham (*P *< 0.0001) representing smoother running of the delivery process after initial teething problems. For those undergoing SNP genotyping, this was 64.4%. Many of the SNP-PRS results were delayed due to delays in women submitting their saliva sample from home.

**Table 4:** Results delivered within 8 weeks for those in the main study without DNA testing.

Site	Within risk feedback timeframe	Outside of risk feedback timeframe	Total	Proportion within 8 weeks	*P* value
Withington^a^ phase 1	118	111	229	51.53%	<0.0001
Trafford/Wythenshawe van^a^ phase 1	189	71	260	72.69%	<0.0001
Oldham^a^ phase 2	152	4	156	97.44%	Reference
East Lancashire	203	77	280	72.50%	
East Cheshire	882	204	1086	81.22%	
Total	1544	467	2011	76.78%	

^a^Greater Manchester sites.

### Change in recruitment materials

We were unable to assess the impact of all the changes to recruitment strategy as these were also affected by changing from Phase 1 to Phase 2 and several COVID-19 related changes. This included decreeing that all women wishing to have screening mammography book their own appointments. As BC-Predict was predicated on the timing of the mammogram, only those booking appointments were approached in Phase 2. However, a significant amendment was instituted at the end of March 2021 which included: amendments to the consent form and participant information sheet in order to improve uptake to BC-Predict by highlighting that a paper consent form was available and adapting the participant information sheet to reflect recommended changes made by a patient involvement group and patient representative-(FH). Other substantial changes were made to increase recruitment uptake, such as inviting women from previous studies (PROCAS-1) in order to obtain an updated risk score. These changes appeared to further increase uptake from around 16% overall to 18% (Supplementary Table 3; *P* = 0.03).

## Discussion

The present research is the first to our knowledge to report the feasibility and impact of real time breast cancer risk feedback based on mammographic density and standard risks on a population basis. The present research has found that it is feasible to set up risk estimation including automatic measures of mammographic density in several NHSBSP sites and provide risk feedback in a timely manner. Uptake rates to the study overall were generally low, and with lower BC-Predict uptake amongst lower SES (socioeconomic status) women. However, BC-Predict uptake rates were highly sensitive to changes in recruitment strategies (Table 2). Importantly, BCPM was considerably higher than has been found in previous research studies.

We have shown that it is feasible to provide risk feedback within a timeframe of 6–8 weeks from a woman undergoing a planned mammographic screen, despite major disruptions during the COVID-19 pandemic. There was clearly a ‘learning’ curve with Phase 2 recruitment from the Greater Manchester sites having very high return rates within 8 weeks (97.5% Table 4). Importantly, we were able to incorporate an automatic measure of mammographic density (Volpara^TM^) into the risk algorithm without the need for direct human involvement. This did require fitting of an aerial to the mobile screening units, which had to be removed and refitted each time the vans moved site. Furthermore, the addition of a SNP-PRS is also feasible, although our plans to roll this out more widely were hampered by the COVID-19 pandemic.

Uptake of the offer of risk estimation in BC-Predict (*n* = 2472) was lower than in PROCAS-1 (*n* = 56,998) [10]. This may be partly a result of the pandemic and also the offer of an online rather than a paper questionnaire. Nonetheless we have demonstrated that even in the lowest recruitment site, we were able to boost recruitment from only 9.1% of those attending for screening to 63.2% if a health practitioner was on site to explain the study and offer a paper questionnaire. This has important implications for implementation as it appears that the use of an online system may deter women from participation. Feedback from women to the health practitioner suggests that this is not just down to inability to use the Internet, but also a suspicion about providing personal information in this way. Age was unlikely a factor as the main target groups were under the age of 63 years. Having to use paper questionnaires will have cost implications for any roll out as in order to prevent further health inequalities, it will be necessary to not only provide personnel to explain the rationale for risk stratification, but also to input some of the risk information from paper questionnaires. The 14% level of non-white British who consented to BC-Predict does nonetheless reflects a higher minority ethnic proportion than the 9% in PROCAS-1 [10]. We have shown the expected lower uptake in higher deprivation areas-(Table 1), but also that this can potentially be addressed by use of a health practitioner and provision of the option of a paper questionnaire. That is, even amongst women from lower SES backgrounds, the obstacles to recruitment can be overcome with appropriate recruitment strategies, rather than reflecting any intrinsic resistance to risk stratified screening per se. There was no evidence for lower uptake of mammography screening when BC-Predict was offered but as the original counterbalanced design was not possible and rates were affected by both COVID-19 and changes to the screening invitation, it was difficult to obtain direct comparisons. The pandemic resulted in dramatic changes to the study design and recruitment, and made it very difficult to assess both the impact of BC-Predict on uptake of screening in general and the impact of changes to the recruitment strategies [14].

While overall uptake of risk appointments was also lower than expected with only just over 40% of those at high risk taking up an appointment, the uptake of BCPM amongst attendees was exceptionally high at 77.5%. This is markedly higher than the average 10–11% uptake in our FHRPC utilising the same clinicians [29, 30], in other high risk population settings [29] and in eligible women in the USA at <8% [31]. This may reflect a greater willingness to do something active about their risk in women newly identified as being at increased risk compared to women in the FHCRPC who may have known their risk, through their family history, for many years. Uptake was significantly higher in high risk than moderate risk women both of a risk appointment (*P* < 0.0001) and of BCPM (*P* < 0.0001) as expected from our previous work [10, 26, 27]. Most women who attended also enrolled in additional screening-(Table 2) although this often required them paying for this as those over 60 were not entitled to be offered NHS screening even if high risk [6]. Even with the relatively small overall numbers, the mean 10-year risk of 11% for high-risk and 6% for moderate-risk women meant the potential prevention of 9–10 breast cancers over 20 years (22% from 62/2 = 6.8 + 12% from 43/2 = 2.6).

We have shown separately that in both questionnaire and qualitative nested sub-studies that there is no evidence of adverse effects on anxiety beyond transient cancer worry [26]. Further, evidence from interviews with HCPs [27] illustrates that the practice of delivering risk-stratified screening was much less burdensome than healthcare professionals anticipated prior to delivery [14].

There are some limitations to the present research. The study was not randomised and was dramatically impacted by the COVID-19 pandemic with uptake of the study and of risk appointments in those identified at moderate or high risk almost certainly affected. As such, generalisability of the results may need to be reassessed after the results from two large randomised trials [32, 33] are available. We were unable to use the original counterbalanced design, which would have allowed better estimation of differences between BC-Predict and NHSBSP rates and could not answer the question of whether there were lower rates for screening uptake (although there was no evidence of this in PROCAS-1 [10]). Several recruitment methods were used and adapted throughout the study which allowed us to examine uptake rates through these different methods, but this will have influenced the uptake figure. There is also a need for a study on cost-effectiveness; a subsequent analysis is planned. There are also a number of strengths: this is the first study to examine what happens when risk stratification happens in real time. We have shown that population risk feedback is feasible in a national screening programme (including an automated breast density input) and change in recruitment strategies and in particular explanation of the purpose by a health practitioner hugely increases uptake. Evaluation of the feedback letters which were co-produced with participants in PROCAS-1 [16] is being reported in a sister study. The study is the first to our knowledge to provide the impact of offering real time risk provision in a national screening programme. The study had a diverse sample across a number of high deprivation areas. An adaptive design facilitated the high uptake of preventive medicine in those eligible. It also addresses a number of research gaps identified in breast cancer risk stratification [23, 34]. There are a number of other important initiatives in progress including the WISDOM [32] and MyPEBS [33] studies, but both are still open to recruitment and have not yet reported results of risk feedback.

In conclusion, we have shown that breast cancer risk stratification can be done as part of routine NHSBSP delivery and supports the uptake of preventive medicines for women at high risk of breast cancer. For uptake, a simple letter invitation does not work and real time risk assessment is feasible if it does not include SNPs. Ideally, all women at moderate and high risk should have at least telephone feedback to further improve uptake of preventive medication. There is a need to consider how to increase uptake especially amongst lower SES and ethnic minority women, to avoid exacerbating inequalities. The present study provides important information on likely uptake rates for risk estimation, risk consultations and preventive options that are necessary to inform subsequent economic analyses of the healthcare costs and patient consequences (benefits and harms) in BC-Predict and of introducing risk stratification into the NHSBSP.

## Supplementary information

Supplementary figure legends

Supplementary tables 1-3

## Data Availability

Not applicable, as it is a protocol paper.
