# Correction To: Quantifying the effects of risk-stratified breast cancer screening when delivered in real time as routine practice versus usual screening: the BC-Predict non-randomised controlled study (NCT04359420)

**DOI:** 10.1038/s41416-023-02273-3

**Published:** 2023-04-24

**Authors:** D. Gareth Evans, Lorna McWilliams, Susan Astley, Adam R. Brentnall, Jack Cuzick, Richard Dobrashian, Stephen W. Duffy, Louise S. Gorman, Elaine F. Harkness, Fiona Harrison, Michelle Harvie, Andrew Jerrison, Matthew Machin, Anthony J. Maxwell, Sacha J. Howell, Stuart J. Wright, Katherine Payne, Nadeem Qureshi, Helen Ruane, Jake Southworth, Lynne Fox, Sarah Bowers, Gillian Hutchinson, Emma Thorpe, Fiona Ulph, Victoria Woof, Anthony Howell, David P. French

**Affiliations:** 1 NIHR Manchester Biomedical Research Centre, Manchester Academic Health Science Centre, Central Manchester University Hospitals NHS Foundation Trust, Manchester, England; 2 The Nightingale and Prevent Breast Cancer Centre, Manchester University NHS Foundation Trust, Manchester, M23 9LT England; 3 Manchester Breast Centre, Manchester Cancer Research Centre, University of Manchester, 555 Wilmslow Road, Manchester, M20 4GJ England; 4 Genomic Medicine, Division of Evolution and Genomic Sciences, The University of Manchester, St Mary’s Hospital, Manchester University NHS Foundation Trust, Oxford Road, Manchester, M13 9WL England; 5 Manchester Centre of Health Psychology, Division of Psychology and Mental Health, School of Health Sciences, University of Manchester, Coupland Street, Manchester, M13 9PL England; 6 Division of Informatics, Imaging and Data Sciences, School of Health Sciences, University of Manchester, Manchester, England; 7 Centre for Prevention, Detection and Diagnosis, Wolfson Institute of Population Health, Queen Mary University of London, London, England; 8 East Lancashire Hospitals NHS Trust, Royal Blackburn Hospital, Haslingden Road, Lancashire BB2 3HH Manchester, England; 9 NIHR Greater Manchester Patient Safety Translational Research Centre, University of Manchester, Manchester, M13 9PL England; 10 Patient representative, Manchester, England; 11 Research IT, IT Services, University of Manchester, Manchester, M13 9PL England; 12 Department of Medical Oncology, The Christie NHS Foundation Trust, Wilmslow Road, Manchester, M20 4BX England; 13 Manchester Centre for Health Economics, Division of Population Health, Health Services Research & Primary Care, School of Health Sciences, University of Manchester, Manchester, M13 9PL England; 14 Primary Care Stratified Medicine research group, Centre for Academic Primary Care, University of Nottingham, University Park, Nottingham, NG7 2RD England

**Keywords:** Genetics research, Health services, Epidemiology

Correction to: *British Journal of Cancer* 10.1038/s41416-023-02250-w, published online 01 April 2023

The article Quantifying the effects of risk-stratified breast cancer screening when delivered in real time as routine practice versus usual screening: the BC-Predict non-randomised controlled study (NCT04359420), written by D. Gareth Evans, Lorna McWilliams, Susan Astley, Adam R. Brentnall, Jack Cuzick, Richard Dobrashian, Stephen W. Duffy, Louise S. Gorman, Elaine F. Harkness, Fiona Harrison, Michelle Harvie, Andrew Jerrison, Matthew Machin, Anthony J. Maxwell, Sacha J. Howell, Stuart J. Wright, Katherine Payne, Nadeem Qureshi, Helen Ruane, Jake Southworth, Lynne Fox, Sarah Bowers, Gillian Hutchinson, Emma Thorpe, Fiona Ulph, Victoria Woof, Anthony Howell and David P. French, was originally published electronically on the publisher’s internet portal on 1^st^ April 2023 without open access. With the author(s)’ decision to opt for Open Choice the copyright of the article changed on 4^th^ April 2023 to © The Authors 2023 and the article is forthwith distributed under a Creative Commons Attribution 4.0 International License, which permits use, sharing, adaptation, distribution and reproduction in any medium or format, as long as you give appropriate credit to the original author(s) and the source, provide a link to the Creative Commons licence, and indicate if changes were made. The images or other third party material in this article are included in the article’s Creative Commons licence, unless indicated otherwise in a credit line to the material. If material is not included in the article’s Creative Commons licence and your intended use is not permitted by statutory regulation or exceeds the permitted use, you will need to obtain permission directly from the copyright holder. To view a copy of this licence, visit http://creativecommons.org/licenses/by/4.0/.

