# Supplementary material for: Quantifying the effects of risk-stratified breast cancer screening when delivered in real time as routine practice versus usual screening: the BC-Predict non-randomised controlled study (NCT04359420)
Source: Br J Cancer. 2023 Apr 1;128(11):2063–71. doi: 10.1038/s41416-023-02250-w (PMC10066938; doi:10.1038/s41416-023-02250-w)
Supplement: Supplementary file 1 — Supplementary figure legends [file 41416_2023_2250_MOESM1_ESM.docx]

Supplementary Materials (results letters)

1. Above average results letter
2. High risk results letter
3. Average risk results letter
4. Below average results letter
