# Supplementary material for: Quantifying the effects of risk-stratified breast cancer screening when delivered in real time as routine practice versus usual screening: the BC-Predict non-randomised controlled study (NCT04359420)
Source: Br J Cancer. 2023 Apr 1;128(11):2063–71. doi: 10.1038/s41416-023-02250-w (PMC10066938; doi:10.1038/s41416-023-02250-w)
Supplement: Supplementary file 2 — Supplementary tables 1-3 [file 41416_2023_2250_MOESM2_ESM.docx]

**Supplementary table 1**: Timeline of BC-Predict with sites and changes to recruitment

| Phase or changes to protocol | Dates | Sites | Changes |
| --- | --- | --- | --- |
| Pilot phase | June-August 2019 | Withington Community |  |
| Phase 1 | Sept 2019-August 2020 | Withington Community, Trafford Van | Active main study recruitment |
| Covid-19 delay | March 21-Aug-1^st^ 2020 | Withington Community, Trafford Van | Pause to main study recruitment |
| Phase 1 | November 2019-March 2020 | Withington Community | On site practitioner recruitment  Saliva study recruitment |
| Phase 2 | Sept 2020 -July 2021 | Oldham Greater Manchester, two East Cheshire sites | Active main study recruitment |
| Phase 2 | February -July 2021 | two NE Lancashire | Active main study recruitment |
| Changes to protocol in Phase 2 | March 2021 | Oldham Greater Manchester, two East Cheshire sites, two East Lancashire | Amendments of the consent form and patient information sheet (PIS) in order to improve uptake to the main study by highlighting a paper consent was available and adapting the PIS to reflect recommended changes made by a PPI group and patient representative. Other substantial changes were made to increase recruitment uptake, such as inviting women from previous studies in order to obtain an updated risk score. |

**Supplementary Table 2:** Family History of Breast Cancer related to Site and whether consented to DNA testing

| **Site** | **Total with 1^st^ degree FH** | **Total** | **%** | **P value** |
| --- | --- | --- | --- | --- |
| Withington no SNPs | 77 | 275 | 28.00% | reference |
| Withington with SNPs | 22 | 130 | 16.92% | <0.0001 |
| Oldham no SNPs | 49 | 156 | 31.41% | reference |
| Oldham with SNPs | 21 | 100 | 21.00% | 0.08 |
| East Lancs | 43 | 280 | 15.36% |  |
| East Cheshire | 202 | 1086 | 18.60% |  |
| Trafford/Wythenshawe van | 72 | 445 | 16.18% |  |
| Total | 486 | 2472 |  |  |

**Supplementary Table 3:** Increase in Recruitment to BC-Predict after implementing changes to the invitation letter and information sheet

| Month | invited | Recruited | Proportion recruited | p value |
| --- | --- | --- | --- | --- |
| November 20 | 774 | 96 | 12.40% |  |
| December 20 | 533 | 96 | 18% |  |
| January 21 | 700 | 151 | 21% |  |
| February 21 | 1015 | 147 | 14.4 |  |
| Total | 3022 | 490 | 16.21% | Reference |
| **Amendment implemented** |  |  |  |  |
| March 21 | 1505 | 283 | 18.80% |  |
| April 21 | 1271 | 221 | 17.30% |  |
| May 21 | 1152 | 209 | 18.10% |  |
| Total | 3928 | 713 | 18.15% | 0.0348 |
